# Dystrophic calcification within biologic graft occurring with use of calcium sulfate antibiotic beads masquerading as an enteric fistula

**DOI:** 10.1093/jscr/rjac270

**Published:** 2022-06-16

**Authors:** Samuel Minor, Judy Rowe, Marius Hoogerboord

**Affiliations:** Department of Surgery and Critical Care Medicine, Dalhousie University, Halifax, Nova Scotia, Canada; Department of Radiology, Dalhousie University, Halifax, Nova Scotia, Canada; Department of Surgery, Dalhousie University, Halifax, Nova Scotia, Canada

## Abstract

The rare (<2%) development of calcium deposits in soft tissue, known as dystrophic calcification (DC) with the use of Stimulan® (Biocomposites Ltd, Wilmington, NC) absorbable, calcium sulfate antibiotic beads (CSABs) in the setting of orthopedic surgery has previously been described. However, the use of CSAB in hernia repair is relatively novel and its association with the development of DC in this setting has not been previously reported. We describe a case where DC following abdominal wall reconstruction with CSAB was misinterpreted on CT imaging as an enteric fistula and almost resulted in an unnecessary emergency surgical procedure.

## INTRODUCTION

Complex ventral hernia repair in the setting of contamination is associated with infection rates as high as 46% [1] and has significant attributable morbidity and cost [2]. Consequently, strategies to prevent infection in this setting are of great interest. We have previously reported on the use of Stimulan® (Biocomposites Ltd, Wilmington, NC) absorbable, calcium sulfate antibiotic beads (CSABs) in patients at high risk for infection undergoing abdominal wall reconstruction with reinforcement of the repair with porcine submucosal hernia graft [1]. CSAB are a biodegradable material that locally deliver high levels of antibiotics with minimal systemic absorption. Their use in decreasing or treating infection in a variety of surgical implants has previously been described ^2^ [3, 4], The beads are radiopaque and completely absorb over 3–12 weeks [5]. However, the rare (<2%) development of calcium deposits in soft tissue, known as dystrophic calcification (DC), has been described in the use of CSAB implanted into soft tissue in the setting of orthopedic surgery [5]. The use of CSAB in hernia repair is relatively novel and its association with the development of DC has not been previously reported. This is an important finding to be aware of as we describe a case in which DC was misinterpreted on CT imaging as extra luminal contrast consistent with an enteric fistula.

## CASE REPORT

**Figure 1:**
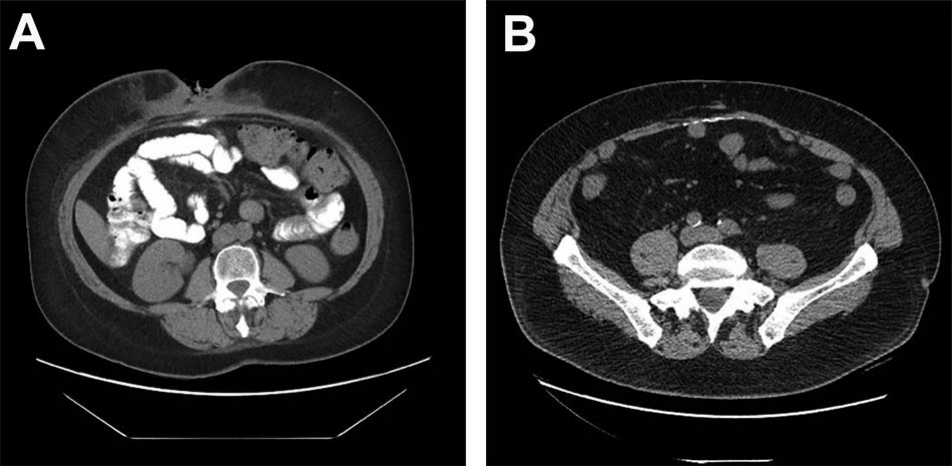
(**A**) DC misinterpreted as extra luminal contrast consistent with a fistula. (**B**) DC distributed horizontally along the hernia graft in a patient where CSAB were used.

A 59-year-old female developed an incisional hernia following an open hiatal hernia repair. She underwent a repair with polypropylene mesh that was complicated by a mesh infection that required partial mesh explantation. Two years later, she re-presented with a recurrent midline incisional hernia. Her risk factors for infection included prior mesh infection, smoking and a body mass index (BMI) of 35. The hernia fascial defect measured by CT was 15 cm wide with a length of 10 cm. Pre-operatively, the patient quit smoking. One month prior to surgery, she underwent Botox injection of the abdominal muscles to facilitate fascial reapproximation. At surgery, bilateral anterior component separation with release of the external oblique muscles was performed, the fascia was closed primarily and the repair was reinforced with a 20 cm×30 cm porcine submucosa hernia graft (Biodesign® Cook Medical Ltd, Indiana, Unites States), placed intraperitoneally and anchored circumferentially with interrupted full thickness absorbable, monofilament sutures (#1 PDS® Medtronic Ltd., Dublin, Ireland). Stimulan 20 cc CSAB infused with vancomycin 4 gm and gentamicin 240 mg were utilized, with half of the beads placed on top of the mesh, below the fascia and the other half on top of the fascia in the subcutaneous space. We left a drain below the fascia on the mesh and one on each side deep to the subcutaneous flaps. We approximated the skin with a running 4–0 absorbable monofilament suture (Monocryl® Ethicon, Georgia, USA) and applied a Prevena™(KCI Technologies Ltd, San Antonio, Texas) negative pressure dressing over the incision. Operative time was 3 h 11 min. The patient had an uncomplicated early recovery and was discharged home on postoperative day 5. The patient returned 2 weeks later with a superficial wound dehiscence that was managed with negative pressure wound dressing. Five months later, the wound had still not completely granulated in and a CT scan demonstrated a (6×8 cm) abscess between the fascia and the mesh. The fascial closure remained intact and the abscess was managed by percutaneous drainage. Cultures demonstrated *Candida albicans* and the patient was treated with an 8-week course of fluconazole. During this time the percutaneous drainage ceased and the drain was removed. The patient presented to the emergency department 7 months later with worsening abdominal pain and a CT scan was performed (Fig. 1A). The report noted ‘an abnormal collection of oral contrast lying within the base of the anterior midline indicating there is a fistulous track communicating with an adjacent small bowel segment’. On the basis of the CT findings, she was advised by the on-call surgeon that she needed emergency surgery, mesh explantation and segmental small bowel resection. However, there was no clinical evidence of enteric discharge through the wound and the patient remained well with no fever and no elevation in white blood cell count. In consultation with the original surgeon, it was decided to forego emergency operative exploration and continue conservative management. Two months later, the subcutaneous wound had still not completely granulated and an exploration and debridement under general anesthesia was performed that failed to demonstrate an enteric fistula. The wound was eventually managed by local skin flap advancement.

## DISCUSSION

Only two cases of DC in incisional hernia repair have been previously described, both involving polypropylene mesh [6–8]. Both these cases described the calcium deposited in a vertical manner along the linea alba. Although it is rare for DC to be clinically relevant, it has been estimated that some degree of calcinosis occurs in up to 25% of laparotomies, usually as a punctate deposit in the midline, or along suture points of the anterior or posterior rectus sheath [9]. In our series of 23 patients undergoing incisional hernia repair with porcine submucosa hernia graft and CSAB, 6 of the 11 patients (55%) who had a post-operative CT had evidence of DC occurring horizontally along the hernia graft insertion plane (Fig. 1B).

In general, the risk factors for developing DC include infection, trauma, burns, neurologic injury and arthroplasty [10]. We postulate that the higher rate of DC observed in patients in whom CSAB was used in combination with a porcine submucosa graft could be secondary to the high calcium levels created by the dissolving CSAB at the time when fibroblast ingrowth into the graft is occurring.

## CONCLUSION

This is the first reported case of DC associated with CSAB combined with a porcine submucosa hernia graft. This case highlights that the atypical horizontal linear calcifications can be misconstrued as extraluminal contrast on CT. Considering the high frequency in which calcinosis is being observed in this patient population, it is important to be aware of this phenomenon in the post-operative evaluation of this often complex patient population.

## CONFLICT OF INTEREST STATEMENT

S.M. has received research funding by COOK medical for a different study. S.M. has also received speaking honorarium from COOK. J.R. and M.H. have no reported conflict of interest.

## FUNDING

None.

## CONSENT FOR PUBLICATION

Written informed consent was obtained from the patient for publication of this case report and any accompanying images. A copy of the written consent is available for review by the Editor of this journal.

## ETHICS APPROVAL

All patients involved have provided consent for their medical information to be used in a case report. Research ethics board waived for case report.
